# Emergency Medicine Journal Editorial Boards: Analysis of Gender, H-Index, Publications, Academic Rank, and Leadership Roles

**DOI:** 10.5811/westjem.2020.11.49122

**Published:** 2021-03-02

**Authors:** Daria Hutchinson, Priya Das, Michelle D. Lall, Jesse Hill, Saleh Fares, Faisal Khosa

**Affiliations:** * University of British Columbia, Faculty of Medicine, Vancouver, British Columbia, Canada; † Universirty of Kerala, Department of Computational Biology and Bioinformatics, Kerala, India; ‡ Emory University School of Medicine, Department of Emergency Medicine, Atlanta, Georgia; § University of Alberta, Faculty of Medicine and Dentistry, Edmonton, Alberta, Canada; ¶ Zayed Military Hospital, Department of Emergency Medicine, Abu Dhabi, United Arab Emirates; || Vancouver General Hospital/University of British Columbia, Department of Radiology, Vancouver, British Columbia, Canada

## Abstract

**Introduction:**

Our goal in this study was to determine female representation on editorial boards of high-ranking emergency medicine (EM) journals. In addition, we examined factors associated with gender disparity, including board members’ academic rank, departmental leadership position, h-index, total publications, total citations, and total publishing years.

**Methods:**

In this retrospective study, we examined EM editorial boards with an impact factor of 1 or greater according to the Clarivate Journal Citations Report for a total of 16 journals. All board members with a doctor of medicine or doctor of osteopathic medicine degree, or international equivalent were included, resulting in 781 included board members. We analyzed board members’ gender, academic rank, departmental leadership position, h-index, total publications, total citations, and total publishing years.

**Results:**

Gender disparity was clearly notable, with men holding 87.3% (682/781) of physician editorial board positions and women holding 12.7% (99/781) of positions. Only 6.6% (1/15) of included editorial board chiefs were women. Male editorial board members possessed higher h-indices, total citations, and more publishing years than their female counterparts. Male board members held a greater number of departmental leadership positions, as well as higher academic ranks.

**Conclusion:**

Significant gender disparity exists on EM editorial boards. Substantial inequalities between men and women board members exist in both the academic and departmental realms. Addressing these inequalities will likely be an integral part of achieving gender parity on editorial boards.

## INTRODUCTION

### Background

Emergency medicine (EM) is a rapidly growing and highly competitive medical specialty, with over 4000 residency applicants within the United States alone in 2019.^1^ Despite the fact that medical schools now graduate equal numbers of men and women, EM remains predominantly male, with men representing over 72% of emergency physicians (EP), compared to 65% of physicians across medicine as a whole.^2^ This demonstrable gender gap has decreased over the past several years, with the percentage of female EPs increasing from 22% in 2007 to nearly 30% in 2018.^2^ Despite the increasing proportion of women in EM, there exists an ongoing under-representation within the field of academic EM.^3^–^5^ Recent data from the Association of American Medical Colleges (AAMC) demonstrates that women still represent the minority of departmental and faculty leadership positions within EM; only 11.4% of EM chairs and 19.3% of full professors are women.^6^,^7^ Participation in academia, including peer-reviewed research, is important to the advancement of the profession and would be best served with equitable representation of its constituents.

A recent study examining academic positions held by EPs found that 17% of male academic EPs held the rank of full professor, compared to only 7% of female EPs.^3^ The difference was less prominent but still notable for associate professor positions, with 24% of men holding this position compared to 19% of women.^3^ Men held more than twice the number of chair and vice chair positions, at 10% vs only 4% of women.^3^ Furthermore, this study found significant discrepancies in income, with the mean salary of academic female EPs $19,418 less than males, even when potentially confounding factors such as experience, clinical hours, and training were accounted for.^3^

### Importance

Previous studies have found substantial gender imbalances within academic disciplines, professional societies,^8^–^10^ and editorial boards of medical journals across a wide variety of medical specialties.^11^–^20^ In 2011 only 17.5% of board members and 15.9% of editors-in-chief across 60 major medical journals were found to be women.^16^ Within EM these numbers are even lower, with women comprising only 13.2% of board members and 3.6% of editors-in-chief in 2010.^21^ Appointment to an editorial board is viewed as a position of influence or eminence and is often sought by both male and female candidates; accepting this premise, it is unfortunate the women have been so persistently under-represented.^16^–^19^,^22^

### Goals of This Investigation

We set out to examine and characterize gender disparities within EM editorial boards, using board demographics to assess whether any progress has been made over the past decade. Our primary outcomes measures were the proportion of male and female board members on each journal’s editorial board, as well as academic achievement of these members based on h-index, departmental/academic rank, number of publications, number of citations, and total publishing years.

## METHODS

### Study Design and Setting

This was a retrospective, descriptive study examining all doctor of medicine, doctor of osteopathic medicine (MD/DO) or international-equivalent board members on high-ranking EM journals. Data collection took place from January–May 2019. The study did not require institutional review board approval as all data obtained were publicly available on journal websites and databases.

**Population Health Research Capsule**

What do we already know about this issue?

Previous studies have found large differences in the gender distribution of academic emergency medicine and emergency medicine journal editorial boards.

What was the research question?

What is the proportion of men and women on EM editorial boards, and has this number changed significantly over the past decade?

What was the major finding of the study?

There were more men than women on all EM journal boards examined. There has been little progress in this regard over the past 10 years

How does this improve population health?

Although the proportion of female EM doctors has increased, women are still vastly outnumbered on EM editorial boards. This study is an important step in addressing this complex issue.

### Selection of Journals

Our study included EM medical journals with an impact factor of 1 or higher based upon the 2017 InCite Journal Citation Reports by Clarivate Analytics. Using these criteria, a total of 17 journals were included (Table 1). One journal, *Emergency Medicine Clinics of North America*, was excluded because their editorial board is temporary, with different members overseeing each issue.

### Measurements

The primary outcome of our study was the number of women and men physicians on selected journal boards. Secondary outcomes included department/academic rank, total number of publications and citations, and active publishing years, as these are metrics that are often used as a measure of academic success.^23^ The h-index, a score calculated based on an author’s number of publications and number of citations per publication was also included, as this is often used as a measure of research productivity.^23^

Gender was recorded as male, female, or unknown. Academic rank was coded as Dean, Assistant dean, professor, associate professor, assistant professor, instructor, or “none” if no academic rank was held. Those with emeritus or honorary standing were placed in the category of “other.” Departmental leadership position was coded as chair, vice chair, director, associate director, or assistant director. Director positions included medical, residency, or clerkship directors.

### Data Extraction

The inclusion criteria were active (non-emeritus) editorial board members holding a medical degree (MD, DO, or an international equivalent). We excluded board members for whom gender could not be determined, those that could not be found in Elsevier’s SCOPUS database, or those for whom publicly available information on gender or academic/departmental rank was absent. Information on each editorial board member’s gender, academic rank, and departmental leadership position were elicited through journal webpages, press releases, or from university and hospital directories. Their full names were cross-checked by a Google search to minimize inaccuracies when extracting bibliometrics from Scopus. Gender was determined by a single author via descriptors (he/him, she/her) on journal, university, or hospital webpages or press releases. If no such descriptor could be found, the board member was excluded. We collected editorial board members’ h-index, active publishing years, number of publications, and total number of citations via Elsevier’s SCOPUS database. If an author had multiple entries in SCOPUS, the entry with the higher h-index was used. All data were collected between January–May 2019.

### Analysis

We performed statistical analysis using SPSS version 25.0 (IBM Corp, Armonk, NY). Gender differences were represented as mean/median and percentages. Academic ranks and editorial positions were also represented as mean/median. We performed Pearson’s correlation and Kruskal-Wallis test to deduce the relationship among the bibliometric study variables.

## RESULTS

### Characteristics of Study Subjects

A total of 918 board members were found on the included journals’ editorial board listings. We excluded 137 of these from the study based on the following: non-MD (65); inability to obtain adequate information on gender or lack of information on SCOPUS (59); the board member was deceased at the time of data collection (1); or the board member held an honorary position on the journal board only, ie, emeritus (12). Of those who were excluded, 41.6% were male, 21.9% were female, and 36.5% were of unknown gender. Overall, 781 board members were included in the study, 682 of whom were male and 99 female.

### Main Results

**Figure 1:**
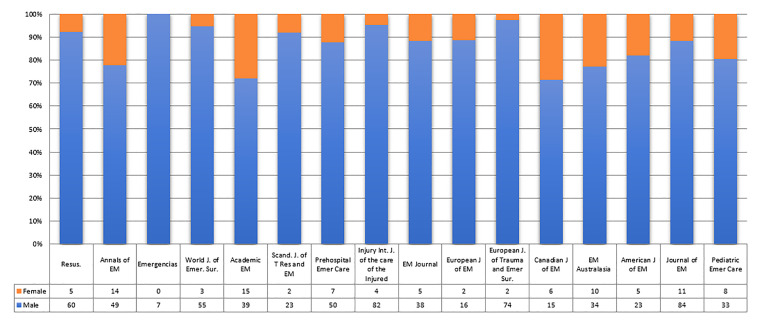
Male and female editorial board members on individual journals. *EM*, emergency medicine.

**Figure 2:**
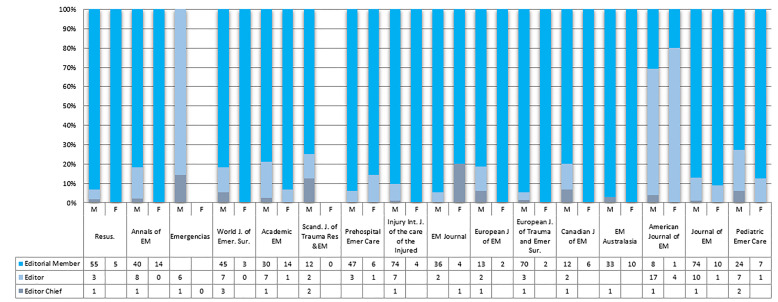
Males and females holding senior positions on editorial boards. *EM*, emergency medicine.

There is a higher number of male editorial board members, with 87.32% (682/781) of editorial board members being male and 12.67% (99/781) female. Figure 1 shows that there is a greater proportion of male to female editorial board members across all selected journals. Senior positions (editor or editor-in-chief) on the editorial boards are more often held by men than women (13.18 %; 103/781 vs females 1.15%; 9/781) (Figure 2).

**Figure 3:**
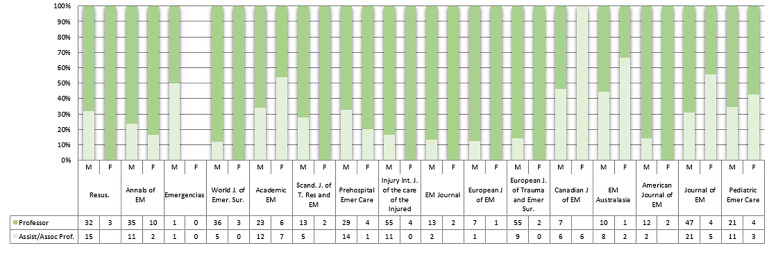
Proportion of male and female editorial board members holding full professor, associate professor, and assistant professor ranks. *EM*, emergency medicine.

**Figure 4:**
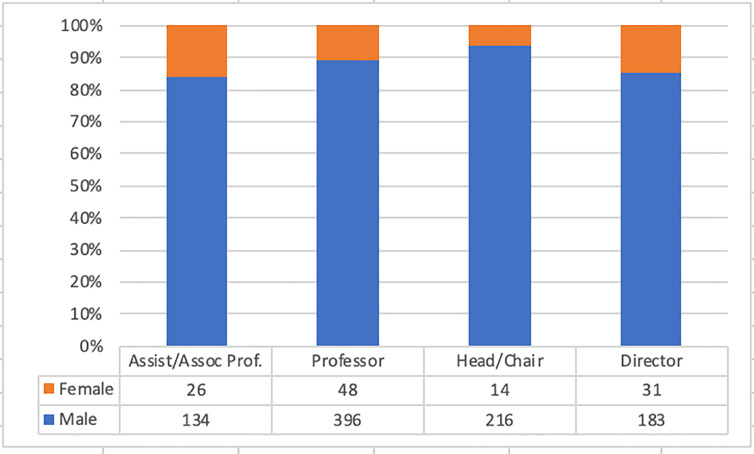
Departmental and academic rank of male vs female editorial board members.

A greater proportion of men than women is seen across all academic ranks (Figure 3) and increasingly so at higher leadership positions (Figure 4). More female editorial board members are assistant or associate professors (26 of 99 [26.3%] vs 134 of 682 [19.6 %]) and more male editorial members are full professors (396 of 682 [58.1%[ vs 48 of 99 [48.5 %]), but the difference is not significant (chi square *P* = 0.17) as seen in Figure 4. Higher departmental ranks are more often held by men. More department heads/chairs are males (216 of 682 [31.7%] vs 14 of 99 [14.1%]); however, a higher proportion of women are directors (31 of 99, 31.6% of women vs 183 of 682, 27.2% of men). This difference is statistically significant with chi-square *P* = 0.004 (Figure 4).

Males have a higher mean h-index (23.8 vs 16.70; *P* <0.0001) and higher mean total citations (3696.41 vs 1670.9; *P* <0.0001). Additionally, males have significantly more publishing years compared to females (28 vs 18; *P* = 0.00051) (Table 2). Bivariate analysis indicates that the number of publishing years are not a predictor of h-index (*P* = 0.12). There were no significant differences between h-indexes of lower and higher academic ranks.

## DISCUSSION

Our study found that significant disparity exists within EM editorial boards. Across all journals included, women physicians on editorial boards were the vast minority, and were far less likely to hold the title of dean or full professor, or prominent departmental positions such as chair. Male editorial board members possessed higher h-indices, total citations, and more publishing years than their female counterparts. These results show that little progress has been made over the last decade: an analysis of 10 high-ranking EM journals by Miro et al in 2010 found women comprised 13.2% of editorial boards, compared to 12.7% in our study. With the percentage of female EPs increasing steadily over the past 10 years,^2^ one would have expected EM editorial boards to experience similar changes in demographics, or at the very least an upward trend, but that was not the case.

Based on 2019 AAMC data, women represent 37.6% of academic EM faculty, 19.3% of professors, and 11.4% of EM chairs. Our study found lower rates of women on editorial boards (12.7%) and who were identified as professors (10.8% of professors) and as chairs (6.1% of chairs/department heads). Unfortunately, this data shows that despite the increased presence of women within academic EM,^7^ there has not been an increase in the number of women represented on editorial boards.

Poor female representation on editorial boards has also been noted in numerous other specialties, including those with relatively high proportions of female physicians. In an extensive review of 60 medical journal editorial boards in 2011, women represented only 17.5% of editorial boards.^16^ Even in journals dedicated to pediatrics and obstetrics, specialties in which female physicians predominate, women were the minority on editorial boards at 30.8% and 26.9%, respectively.^16^ It is clear that the proportion of women in a specialty alone is not responsible for the low numbers seen on editorial boards, and therefore measures aimed simply at increasing the number of female EPs will not be sufficient to address this issue on its own.

The explanation behind this stagnation in progress within EM editorial boards is not entirely clear, and appears not to be solely based on the numbers of women in EM and academic EM. Women in our study were less likely to be full professors or to hold departmental leadership positions, a phenomenon that has been noted by previous research and sometimes referred to as “the glass ceiling” effect.^3^,^4^,^16^,^24^ Our research shows that there is a significant positive correlation between leadership positions and h-index; given that editorial boards often use academic productivity as a selection factor,^8^–^10^,^16^,^18^,^23^ this is a systemic disadvantage for women.

It has been suggested that commitments involved with childbearing and childrearing, predilection for clinical and teaching positions over academic roles, and a lack of mentorship have all impacted the success of women in academic medicine.^16^,^22^,^25^,^26^ Women more frequently hold education and teaching positions,^20^,^24^ which are considered to be of lower value by many institutional promotions and tenure committees.^24^ These roles are critically important but may inhibit those focused on education and teaching from advancing to higher academic ranks at the same rate as those who are focused on research. There are fewer peer-reviewed publication venues for medical educators, which may contribute to the lower number of publications among women in academic EM.

It should also be noted that, owing to a pipeline effect of fewer women entering and remaining in academic EM,^5^,^16^,^24^ the pool of female academic physicians is younger than their male counterparts.^4^ The women in our study had fewer publications, fewer years of publication and lower h-indices, but this may very well be reflective of an earlier career researcher, not someone who is less qualified. Overall, these factors contribute to a scenario in which women are faced with a substantial number of barriers as well as an inequitable selection process for editorial board positions. The creation of female-specific support and mentorship within academic EM is one way of potentially tackling these ongoing disparities,^16^,^22^,^23^,^25^ and is a focus of several initiatives such as the American Academy of Emergency Medicine’s Women in EM Section, the Canadian Association of Emergency Physicians Women in Emergency Medicine Committee, and the Academy for Women in Academic Emergency Medicine. All three of these groups cite support for women in leadership roles and the creation of mentorship opportunities as key goals of their organizations.^27^–^29^

In their 2019 paper, Agrawal et al suggest four core strategies for addressing gender disparities within academic EM: 1) commitment to education on gender bias and its mitigation; 2) ensuring equal resources and opportunities for women as compared to their male counterparts; 3) support for female leadership within EM; and 4) fostering of a workplace culture that allows balance between work and family life.^5^ Importantly, the strategies outlined in their paper are aimed not only at increasing the number of women entering EM, but also at encouraging the retention and support of female EPs as they pass through the various stages, obstacles, and challenges of their careers.

In addition to these measures, we would suggest that journals themselves take on the responsibility to evaluate their editorial boards for adequate representation and set goals for improvement, such as the *Lancet* has done as part of the “#LancetWomen Project.” This initiative, started in December 2017, involved a review of all editorial staff in the *Lancet* group and a subsequent commitment to reaching gender parity on every *Lancet* group journals’ editorial advisory board by 2020.^30^ Although the data for 2020 has not yet been released at the time of this paper’s writing, by February of 2019, 4 out of 14 *Lancet* journals had achieved this goal.^30^

## LIMITATIONS

There are several limitations to our study. Firstly, data collected on board members’ gender, degree, academic rank, and leadership position were obtained through publicly available university and journal websites, or through press releases when the former were unavailable. Although this is a method that has been used and validated by other studies,^10^,^20^,^31^ it is possible that data reported on these sites were either outdated or incorrect. Importantly, this study assumes that gender descriptors used in university/journal biographies or press releases are in line with the board member’s gender identity. It is possible that this is not the case, and in particular may miss board members who do not identify as male or female.

Information on board members’ research, including total number of publications, documents, and citations, total publishing years, and h-index was obtained through Elsevier’s SCOPUS database. When there were duplicate records, the entry with the highest h-index was used. However, some authors may have publications divided between several entries and therefore were not fully credited. Further, researchers could have publications under a different last name. This may impact women more than men, as women are more likely to take their partner’s surname after marriage.

To more accurately compare the proportion of male and female EPs to the proportion on editorial boards, we excluded editorial board members who did not hold an MD, DO, or international equivalent. Of these, 58% were men and 42% were women. Of note, one female editor-in-chief (the *Scandinavian Journal of Trauma*) was excluded from the study based on these criteria. Finally, there were several editorial board members who could not be identified with certainty by using the information provided on the journal’s editorial board website, and others who did not have data in SCOPUS. These individuals were excluded from the study. Presumably, these would contain an equal number of men and women, but as the gender could not be elicited for many of them, the true proportion is unknown.

## CONCLUSION

Representation of women as emergency physicians has increased steadily over the past decade.^2^ This move toward gender parity has not translated to editorial boards of top EM journals, with virtually no change to the proportion of female editorial board members in the past 10 years.^21^ Currently, nearly 30% of EPs in the United States are women,^2^ while only 12.7 % of EM editorial board members are women. Proportional representation is clearly not being achieved, and more needs to be done to address this gap.

## Figures and Tables

**Table 1:** Included journals in gender survey of editorial board membership.

Journal	Impact factor
*Resuscitation*	5.863
*Annals of Emergency Medicine*	5.008
*Emergencias*	3.608
*World Journal of Emergency Surgery*	3.198
*Academic Emergency Medicine*	2.612
*Scandinavian Journal of Trauma Resuscitation and Emergency Medicine*	2.312
*Prehospital Emergency Care*	2.269
*Injury International Journal of the care of the Injured*	2.199
*Emergency Medicine Journal*	2.046
*European Journal of Emergency Medicine*	1.729
*European Journal of Trauma and Emergency Surgery*	1.704
*Canadian Journal of Emergency Medicine*	1.481
*Emergency Medicine Australasia*	1.353
*American Journal of Emergency Medicine*	1.29
*Journal of Emergency Medicine*	1.207
*Pediatric Emergency Care*	1.066

**Table 2:** Publications, citations, and h-index of male and female editorial board members.

	Overall Mean ± SD	Male Mean ± SD	Female Mean ± SD
Number of citations	3467.23 ± 5752.6	3696.41 ± 1679.9	1674.52 ± 2628.5
Years of publication	27.26 ± 67.3	28.53 ± 72.4	18.35 ± 9.75
Number of publications	128.46 ± 140.2	136.35 ± 147.1	68.79 ± 61.4
H-Index	23.15 ± 17.3	23.8 ± 17.8	16.7 ± 12.1

*SD*, standard deviation.
